# The Stacked-Ellipse Algorithm: An Ultrasound-Based 3-D Uterine Segmentation Tool for Enabling Adaptive Radiotherapy for Uterine Cervix Cancer

**DOI:** 10.1016/j.ultrasmedbio.2019.09.001

**Published:** 2020-04

**Authors:** Sarah A. Mason, Ingrid M. White, Susan Lalondrelle, Jeffrey C. Bamber, Emma J. Harris

**Affiliations:** ⁎ Joint Department of Physics, Institute of Cancer Research, London, United Kingdom; † Radiotherapy Department, Royal Marsden NHS Foundation Trust, London, United Kingdom

**Keywords:** Segmentation, Ultrasound-guided radiotherapy, 3-D ultrasound, Uterus, Uterine cervix cancer, Image-guided radiotherapy

## Abstract

The stacked-ellipse (SE) algorithm was developed to rapidly segment the uterus on 3-D ultrasound (US) for the purpose of enabling US-guided adaptive radiotherapy (RT) for uterine cervix cancer patients. The algorithm was initialised manually on a single sagittal slice to provide a series of elliptical initialisation contours in semi-axial planes along the uterus. The elliptical initialisation contours were deformed according to US features such that they conformed to the uterine boundary. The uterus of 15 patients was scanned with 3-D US using the Clarity System (Elekta Ltd.) at multiple days during RT and manually contoured (n = 49 images and corresponding contours). The median (interquartile range) Dice similarity coefficient and mean surface-to-surface-distance between the SE algorithm and manual contours were 0.80 (0.03) and 3.3 (0.2) mm, respectively, which are within the ranges of reported inter-observer contouring variabilities. The SE algorithm could be implemented in adaptive RT to precisely segment the uterus on 3-D US.

## Introduction

**Fig. 1:**
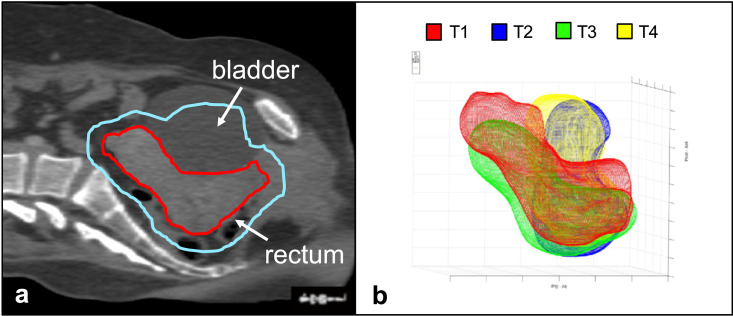
(a) Pre-treatment planning computed tomography image of a cervical cancer patient with the clinical target volume outlined in *red* and the planning target volume outlined in *cyan*. Note that large portions of healthy tissues such as the bladder and rectum are included in the planning target volume. (b) Superimposition of 3-D uterine contours of the cervical cancer patient in (a) derived from ultrasound images taken at four different time points (T1–T4) over the course of radiotherapy treatment. Note the large amount of day-to-day motion and deformation of the uterus over the course of radiotherapy treatment.

The aim of radiotherapy (RT) is to deliver a curative dose to the target tissues (known as the clinical target volume, or CTV) whilst minimising dose to nearby tissues as much as possible to reduce the likelihood of RT-related toxic effects. This is a challenging task when treating cancer of the uterine cervix as the CTV (including the uterus and cervix) undergoes large amounts of day-to-day motion and deformation because of bladder filling, rectal filling and tumour regression (Van de Bunt et al. 2006; Chan et al. 2008; Collen et al. 2010; Bondar et al. 2012; Jadon et al. 2014). To compensate for the positional uncertainty of the uterus–cervix complex (referred to as the uterus for the remainder of this text), the CTV is expanded by 0.6 to 4 cm to form the planning target volume (PTV) (Lim et al. 2011). The generous CTV-to-PTV expansion used in cervical cancer RT improves the likelihood of adequate target coverage at the cost of including large volumes of healthy tissues such as the bladder, rectum and bowel in the PTV (which receives the prescription dose), as illustrated in Figure 1.

If the position of the uterus during RT delivery were known, then the RT treatment plan could be adapted on a daily basis to conform to the CTV. The current gold standard for daily image guidance in RT is cone-beam computed tomography (CBCT), which provides 3-D images of the patient with excellent bony anatomy contrast. Although CBCT does provide some soft tissue contrast and can be used for soft tissue-based treatment verification (*i.e.,* visually assessing whether the uterus is fully contained within the PTV), it is difficult and not always possible to visualise and segment the uterus and other soft tissues in the pelvis because of scatter and reconstruction artefacts (Heijkoop et al. 2014; Langerak et al. 2014; Maemoto et al. 2016; Wang et al. 2016). The excellent soft-tissue contrast of ultrasound (US) makes it a promising alternative to CBCT for localising the uterus prior to RT. Indeed, with the advent of probe-tracking technology, US has been used to guide RT in a variety of anatomical sites, including the prostate, liver, breast and uterus (Fontanarosa et al. 2015). In previous work, we reported that 3-D transabdominal US using the Clarity system (Elekta Ltd., Stockholm, Sweden) can provide high-quality images of the uterus that can be manually segmented with high precision by multiple observers (Mason et al. 2017). However, there is currently no published software tool that can automatically or semi-automatically segment the uterus on 3-D US with sufficient accuracy and speed to be clinically useful. The commercial algorithm available on the Clarity system designed to semi-automatically segment the uterus returns a result in only about 80% of cases and, among these, has variable precision that is dependent on image quality (Mason et al. 2017). Several algorithms for segmenting the uterus in 3-D on magnetic resonance and computed tomography images do exist (Ghose et al. 2015), though it is unlikely that these algorithms would perform well in US images as they rely on modality-specific imaging characteristics such as tissue contrast, field of view and imaging artefacts.

To enable US-guided adaptive RT, a new tool must be developed that can quickly and accurately segment the uterus at the time of treatment on 3-D US images. Segmentation on medical images is a challenging problem, as (i) the shape, contrast and orientation of the target structure with respect to its surroundings vary from person to person, and (ii) every imaging modality has a unique set of characteristics and/or artefacts that can degrade image quality. In the case of US, imaging artefacts such as attenuation (for instance, that caused by bone or gas in the US beam line) and reverberation can obscure target boundaries, create pseudo-boundaries and reduce soft-tissue contrast (Noble and Boukerroui 2006; Wein et al. 2007). Additionally, constructive and destructive wave interference inherent in US imaging gives rise to “speckle” (Burckhardt 1978), which gives US images their characteristic grainy appearance.

**Fig. 2:**
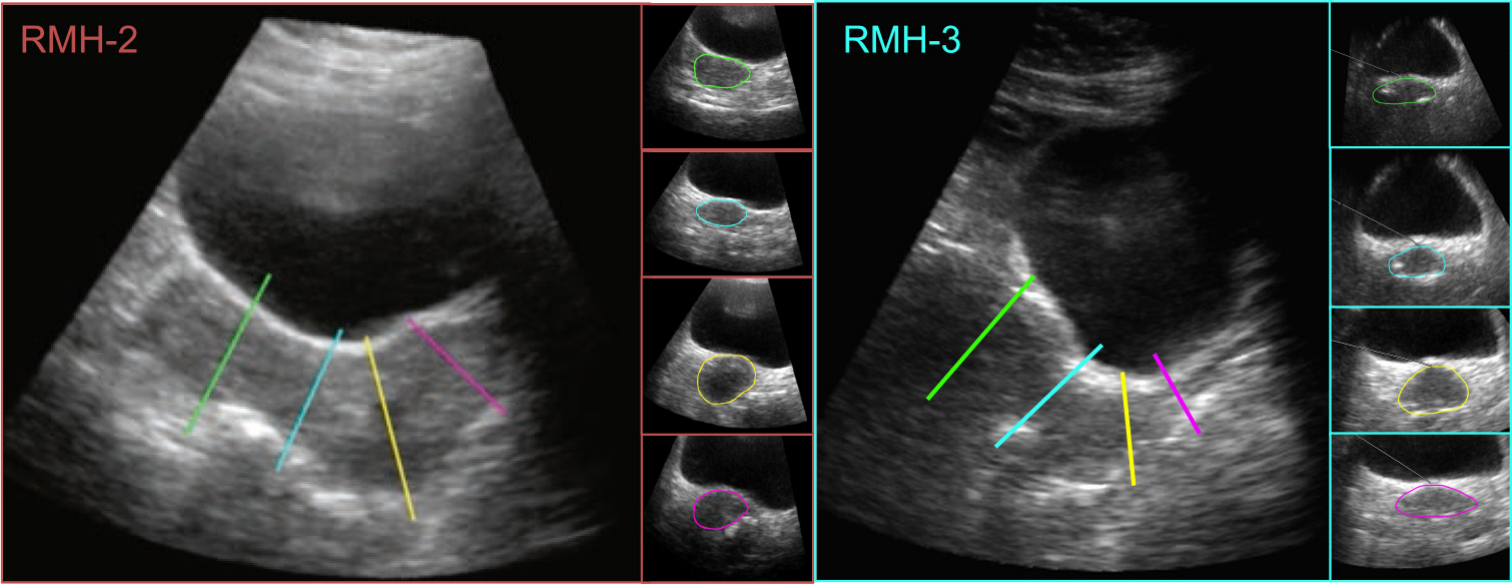
Two patient examples illustrating the elliptical nature of uterine cross sections along the length of the uterus. The position of each cross section is indicated by corresponding colours between the uterine contours in the semi-axial planes and the lines superimposed on the sagittally oriented image.

Parametric shape models can be used to improve the accuracy of segmentation algorithms in the presence of spurious boundaries and image artefacts. In this approach, the target structure is represented as a variation or combination of shapes that can be defined using only a few parameters, such as circles, ellipses and polygons. For example, Gong et al. (2004) used deformable super-ellipses to segment the prostate on 2-D US images with submillimetre accuracy measured in terms of agreement with manual contours. Parametric shape models are a promising solution for segmenting the uteri of cervical cancer patients as uterine cross sections are roughly elliptical, as seen in Figure 2, despite the large anatomical variation between patients.

The aim of this work was to develop an algorithm that could be used to semi-automatically segment the uterus on 3-D images obtained using the Clarity system. A training set of five 3-D US images from five cervical cancer patients was used to represent the uterus as a series of stacked ellipses in a novel segmentation algorithm, which we called the “stacked-ellipse” (SE) algorithm. This algorithm combined conventional boundary detection methods with the prior knowledge that the uterus (i) is darker than its surroundings on US images and (ii) can be represented as ellipses. The SE algorithm was tested in a validation cohort of forty-four 3-D US images from 10 cervical cancer patients by comparing the contours generated by the SE algorithm with corresponding 3-D manual contours.

## Methods

### Data acquisition and patient characteristics

Seventeen patients receiving RT for cervical cancer were considered for this study: 6 from Herlev Hospital, and 11 from the Royal Marsden NHS Foundation Trust (RMH). Ethics approval for these studies was obtained from the De Videnskabsetiske Komiteer and the NHS Research Ethics Committee (Reference No. 15/LO/1438), respectively. Written informed consent was obtained from all patients. Patient characteristics are given in Table 1.

**Table 1:** Baseline characteristics of the patient cohorts from Herlev Hospital and the Royal Marsden NHS Foundation Trust (RMH)

Patient	Age (y)	Weight (kg)	Height (m)	FIGO stage
Herlev-1	40	67.5	1.69	IIIB
Herlev-2	49	63	1.71	IIB
Herlev-3	65	64	1.69	IIB
Herlev-4	59	78	1.68	IIB
Herlev-5	62	103	1.68	IIB
Herlev-6	38	63	1.68	IIB
RMH-1	36	94.1	1.52	IIB
RMH-2	44	62.6	1.47	IIB
RMH-3	50	83	1.71	IIB
RMH-4	65	55.3	1.55	IIB
RMH-5	25	66	1.76	IIB
RMH-6	56	65.5	1.60	IIB
RMH-7	36	62.1	1.75	IIB
RMH-8	57	89.7	1.70	IIB
RMH-9	41	49.5	1.7	IIA
RMH-10	75	67.6	1.59	IIB
RMH-11	71	50.1	1.65	IVA
Mean	51.1	69.9	1.65	-
Standard deviation	14.1	15.0	0.1	-

FIGO = Federation Internationale de Gynecologie Obstetrique (cervical cancer staging criteria).

### Ultrasound scanning protocol

All US data in this study were scan converted 3-D B-mode data acquired with the Clarity system using a hand-held mechanically swept 3-D probe (5-MHz center frequency, Model m4DC7- 3/40, Sonix Series; Ultrasonix Medical Corp., Richmond, BC, Canada). The Clarity system is described elsewhere, but briefly, it is a conventional diagnostic scanner that utilizes infrared tracking technology to determine the position of the US probe (and, hence, the resulting US images) with respect to the isocentre of the treatment room (Lachaine and Falco 2013). At the RMH, the scanning protocol was as follows. One hour prior to the scheduled treatment time, each patient was asked to follow a drinking protocol (void the bladder, drink 350 mL of water in 10 min and then refrain from emptying the bladder until after RT delivery). After the patient had been positioned for treatment by the radiographers, either a trained clinical oncologist or radiographer acquired a 3-D transabdominal US image of the uterus using as little probe pressure as possible. The scanning protocol at Herlev Hospital was similar, but patients were not asked to follow a specific bladder filling protocol, and a medical physicist acquired all US data. Each patient was scanned at multiple time points during her treatment, resulting in a data set of ninety-nine 3-D US image volumes (23 from the 6 patients treated at Herlev Hospital and 75 from the 11 patients treated at the RMH). All US images were resampled onto a Cartesian grid of voxel size 0.58 × 0.58 × 58 mm automatically using Clarity's Automatic Fusion and Contouring workstation.

### Data selection and partitioning

#### Herlev Hospital patients

The highest-quality image from each patient in this cohort comprised an independent training data set for parameterising the uterus as stacked ellipses. The image set from one patient was of substantially poorer quality than the rest. This was therefore removed, so as to minimise the propagation of errors arising from contouring uncertainty, resulting in a training set composed of five 3-D US images from 5 different patients.

#### RMH patients

Images from this patient cohort were used to test the SE algorithm. Of the 75 US images available, the first image acquired from each patient (11 total images) and 40 randomly selected images from the scans performed at later time points were initially evaluated for use in this study. From this data set of 51 US images, a further 7 images were excluded from further analysis because US image quality was too poor to visualise the uterine boundary and thus manually contour (2 from patient RMH-3, 1 from patient RMH-8, 1 from patient RMH-10 and all 3 images from patient RMH-11), resulting in a data set of 44 US images from 10 patients.

## Manual contouring

One experienced observer (S.M.) manually contoured the uterus on the 5 US images from the training set and the 44 images from the validation set using the Clarity Automated Contouring and Fusion workstation. In this workstation, multiple (4–6) 2-D contours were drawn in the sagittal plane spanning the left–right extent of the uterus, and multiple (3–6) 2-D contours were drawn in the axial plane spanning the superior–inferior extent of the uterus. A 3-D contour was then generated by this workstation by interpolating between the contours on the orthogonal planes. This 3-D contour was visually inspected and manually edited (if necessary) to ensure that it conformed to the uterine boundary. Previous work has revealed good agreement between contours drawn by observer S.M. and contours drawn by radiologists and clinical oncologists (Mason et al. 2017). In the Herlev cohort, these contours were used as inputs to train the algorithm. In the RMH cohort, these contours were used as the gold standard for measuring algorithm segmentation accuracy.

### Description of the SE algorithm

**Fig. 3:**
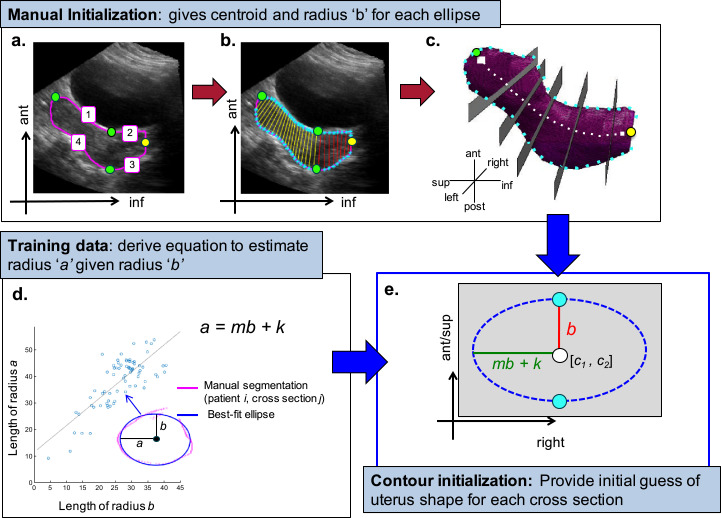
Workflow diagram of the training phase for the stacked-ellipse (SE) algorithm. (a) Manual contour on central sagittal slice in *pink*, with four points placed to divide the contour into segments 1 (top uterus), 2 (top cervix), 3 (bottom cervix) and 4 (top uterus). (b) Anchor points are shown in *cyan. Red and yellow lines* indicate anchor point pairs that define orientation of semi-axial slicing planes, as illustrated in 3-D in (c). Note that in (c), there are fewer slicing planes than would be used for display purposes. (d) Example of best-fit ellipse to manual contour interpolated onto a 2-D semi-axial slice. (e) Relationship between major and minor elliptical axes for all cross-sections and all patients described by a linear fit.

The SE algorithm developed in this work combined a training phase, a 2-D manual initialisation and conventional segmentation techniques based on feature extraction to rapidly segment the uterus on 3-D US images. A single manually initialised 2-D slice in the sagittal plane was used to create a series of 2-D elliptical initialisation contours in semi-axial planes (*i.e.,* axial planes that may have a tilt in the superior–inferior (sup-inf) direction) along the length of the uterus in the sagittal plane (see the *grey rectangles* in Fig. 3c). While the minor axis of each ellipse was defined directly by the manual initialisation step in the sagittal plane, the major axis of each ellipse was estimated using a population-based model derived during the training phase of the SE algorithm. Each 2-D elliptical contour was then deformed according to image features present in the semi-axial planes of the US images such that it conformed to the true uterine boundary, regularised to smooth the contour and correct for outliers, and finally projected into 3-D.

### Training phase

The purpose of the training phase was to develop a model that enabled the estimation of uterine width along semi-axial elliptical cross-sections given the uterine height. The formula for generating an ellipse is given in the equation

(1) $$\frac{{\left(x-c_{1}\right)}^{2}}{a^{2}}+\frac{{\left(y-c_{2}\right)}^{2}}{b^{2}}=1$$

where *c*_1_ and *c*_2_ are the *x*- and *y*-coordinate points of the ellipse centroid, a is the major axis radius (corresponding to anatomical left–right) and b is the minor axis radius.

Three-dimensional manual contours were parameterised as a series of stacked ellipses using the following three steps:

1. **Determine the orientation of semi-axial slices yielding elliptical cross-sections:** Uterine slicing planes should be orientated such that the corresponding uterine cross-sections are approximately elliptical. This could be achieved if these slicing planes were roughly perpendicular to the curved path from the uterine fundus to the base of the cervix (see *dotted white line* on Fig. 3c). This curved path could take any form, depending on where the fundus was with respect to the cervix. Observer S.M. (i) selected the sagittal slice that approximately bisected the uterus into left and right halves, and (ii) placed four landmark points on the uterine contour to split the contour into four segments—top uterus, top cervix, bottom cervix and bottom uterus (see Fig. 3a)—to manually initialize the orientation of the slicing planes. By automatically placing the same number of evenly spaced anchor points on the top and bottom halves of each segment, planes orientated orthogonally or near-orthogonally to both the sagittal image plane and the fundus-to-cervix path were defined by the lines connecting each top–bottom anchor point pair, as illustrated in Figure 3b.
2. **Determine the best-fit ellipse:** The 3-D manual contour was interpolated onto the semi-axial slicing planes generated in the previous step (see *magenta points* in Fig. 3d). The “numerically stable direct least squares fitting of ellipses” method described by Hal and Flusser (1998) was used to find the ellipse that best fit the interpolated manual contour (see *blue ellipse* in Fig. 3d), which enabled the extraction of the corresponding lengths of the major and minor axes (axes a and b respectively). The density of the points constituting the manual contour was sufficiently high to ensure accurate ellipse fitting on every semi-axial slice.
3. **Linear regression:** The axis lengths derived from every cross section j from every patient i in the training set constituted a data point in the model. A linear least-squares fit was used to describe the relationship between the elliptical axes. The resulting equation of the form a = *mb* + *K* was used to estimate the length of axis a given axis *b* of an elliptical uterine cross section in the segmentation phase of the SE algorithm (see Fig. 3e).

### Segmentation phase

**Fig. 4:**
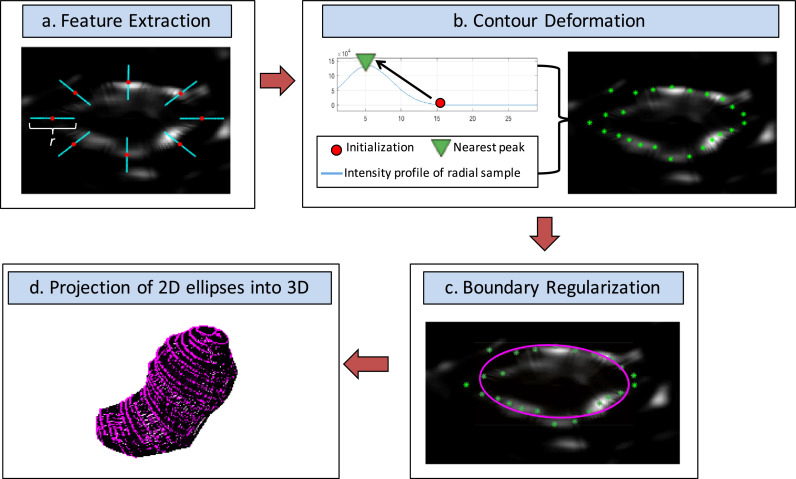
Steps 2–5 of the stacked-ellipse (SE) algorithm workflow. (a) Initialisation ellipse (*red points:* subsampled for visual clarity) superimposed on the directional edge map. *Cyan lines* correspond to uterine boundary search regions. (b) Contour deformation via peak finding. (c) Boundary regularisation via ellipse fitting. (d) Projection of 2-D ellipses into 3-D.

After training, the SE algorithm was able to segment the uterus on an independent data set in the following four steps: (i) manual initialization, (ii) contour deformation, (iii) boundary regularisation and (iv) projection of 2-D contours into 3-D. Each of these steps is described below, and steps 2–5 are depicted in Figure 4.

**Fig. 5:**
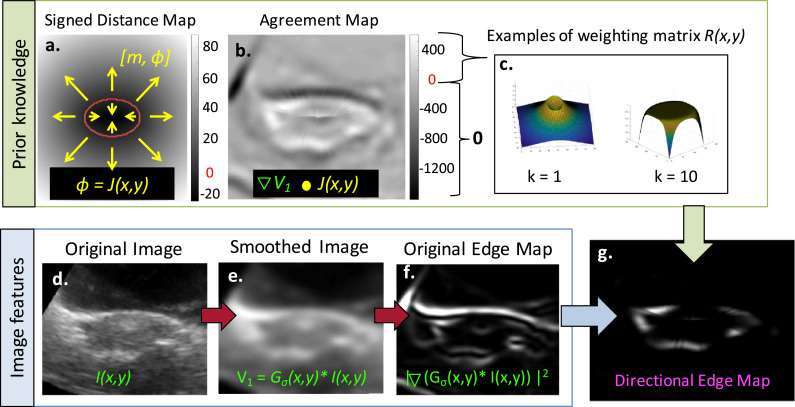
Schematic illustrating how prior knowledge of uterine contrast and shape was combined with image features to generate a directional edge map. (a) A signed distance map was calculated from the initialisation contour shown in *red*. J(x,y) was defined as phase 0 of the signed distance map. The agreement map in (b) was the result of taking the dot product of the gradient of V\ from (e) and J{x, y}: anything <0 was set to zero in the directional edge map. Values on the agreement map that were >0 were then weighted according to R(x, y) to penalise boundaries far from the initialisation contour, as illustrated in (c). R(x,y) had a tuneable parameter k, which determined its descending velocity. Note how the final directional edge map (g) had an enhanced uterine boundary compared with conventional edge maps (f).

1. **Manual initialization:** An observer selected the sagittal slice of the 3D US volume that roughly bisected the uterus into left and right halves, contoured the uterus on that slice and placed four anatomical landmark points on the contour to separate the uterus into top uterus, bottom uterus, top cervix and bottom cervix sections. As in the training phase, evenly spaced anchor points (see cyan asterisks in Fig. 3b) on corresponding top and bottom contour segments were used to define (1) the orientation of the semi- axial image planes that would provide elliptical uterine cross-sections, (2) the minor axis b of each elliptical cross section and (3) the centroid of each ellipse (c_i_, c_2_). An initial guess of parameter a was generated using the linear relationship between a and b determined in the training phase. First-guess elliptical contours were then generated using all of these parameters for every semi-axial plane defined by the anchor points.
2. **Contour deformation:** 2D semi-axial US images were generated by linearly interpolating the original 3D US image into the semi-axial planes defined by the anchor points generated during the Manual Initialization step (see Fig. 5d). The corresponding first-guess elliptical contours were deformed according to boundary information extracted from each 2D semi-axial image. The position of the initialization contours and prior knowledge that the uterus is hypoechoic on US relative to surrounding tissues was used to generate a directional edge map, which lessened the magnitude of, or removed boundaries arising from, negative gradients or boundaries far from the initialization contour. To generate a directional edge map f(x,y) from each 2-D semi-axial image, we used (Le et al. 2015)
  (2) $$f\left(x,y\right)=\left\{ \begin{array}{cc} {\left|\nabla V_{1}\cdot J\left(x,y\right)\right|}^{2}\times R\left(x,y\right) & \text{if}\;\nabla V_{1}\cdot J\left(x,y\right)>0\\ 0 & \text{if}\;\nabla V_{1}\cdot J\left(x,y\right)\leq \;0 \end{array}\right.$$

where V_1_ is the original image *I*(x,y) convolved with a 2-D Gaussian smoothing kernel, *J*(x,y) is the phase of the signed distance map generated using the initialization contour and R(x,y) is a weighting matrix penalizing boundaries far from the initialization contour (see eqn [3]).

Each pixel of the signed distance map was the minimum Euclidean distance between every pixel in the image I(x, y) and the nearest point on the elliptical initialization contour. As illustrated in Figure 5a, points outside of the initialization contour were assigned a positive distance, and points inside of the initialization contours were assigned a negative distance. J(x, y) was used to provide a model for the expected intensity gradient of I(x, y) under the assumption that the uterus was darker than its surroundings. The dot product of the phase component of the gradient of the original image (smoothed by a gaussian kernel–see Fig. 5e) and *J*(x,y) provided a convenient way to quantify the extent to which the true contrast gradient follows the model. Contrast gradients that have the same direction as *J*(*x, y*) were maximized, while contrast gradients that have the direction opposite *J*(*x, y*) were minimized, as illustrated in the agreement map in Figure 5b. In eqn (2), the agreement map corresponds to the term ∇*V*_1_ · *J*(*x, y*). The agreement map was used as a thresholding tool to determine which boundaries to include in the directional edge map. Anything greater than zero (*i.e.,* where the contrast gradient has a phase component along the direction of *J*(*x, y*)) was included, whereas anything less than or equal to zero was set to zero in the directional edge map.

After elimination of spurious boundaries based on gradient, a provisional directional edge map was obtained by squaring the gradient of the agreement map. This provisional directional edge map was modified by a weighting matrix *R*(*x, y*) as outlined in the equation

(3) $$R\left(x,y\right)=\left(1-{\left(\frac{d\left(x,y\right)}{max\left(d\left(x,y\right)\right)}\right)}^{k}\right)\times exp\left(-{\left(\frac{d\left(x,y\right)}{max\left(d\left(x,y\right)\right)}\right)}^{k}\right)$$

where *d*(*x, y*) is the map of distance between every pixel in the image and the nearest point in the provisional contour (*i.e.,* the absolute value of the signed distance map), and k is a tuneable parameter that determines how heavily a boundary is penalised for being located far from the provisional contour. As illustrated in Figure 5c, the smaller the value of *k*, the more heavily boundaries far from the initialisation contour were penalised, as the descending velocity of *R*(*x, y*) was increased. An example of a directional edge map is provided in Figure 5g. The peak brightness of the boundary sections on the directional edge map (Fig. 5g) corresponds to the steepest contrast gradient along the uterine boundary on the original image (Fig. 5d).

To determine where the uterine boundary was on the directional edge maps, the SE algorithm searched for peaks in image intensity on the directional edge map that were nearest to the initialisation points. Specifically, a 1-D intensity profile was extracted from radial samples of length *r* on the directional edge map, and the initialisation contour was moved along that radius to the position of the nearest peak (see Fig. 4). These peak-shifted points formed a provisional 2-D uterine contour for each semi-axial cross section.

3. **Boundary regularisation:** Though the majority of the points constituting the provisional contour were positioned on the true uterine boundary (defined as the position of the steepest contrast gradient along the edge of the uterus on the original image), some either moved to spurious boundaries that remained in the directional edge map or stayed in place if no boundary was present, making the uterine boundary appear jagged. Again relying on the assumption that the uterus had elliptical cross sections, the SE algorithm fitted an ellipse to each provisional contour to smooth the uterine boundary and to mitigate the influence of outliers, as illustrated in Figures 5c and 6. As an ellipse must be fitted to every 2-D semi-axial cross section, the non-iterative “numerically stable direct least squares fitting of ellipses” algorithm (Hal and Flusser 1998) was implemented to minimise the computation time required. The high point density of the 2-D semi-axial provisional contours ensured an accurate fit using this algorithm. These ellipses formed the final contours for each 2-D semi-axial cross section of the uterus.

**Fig. 6:**
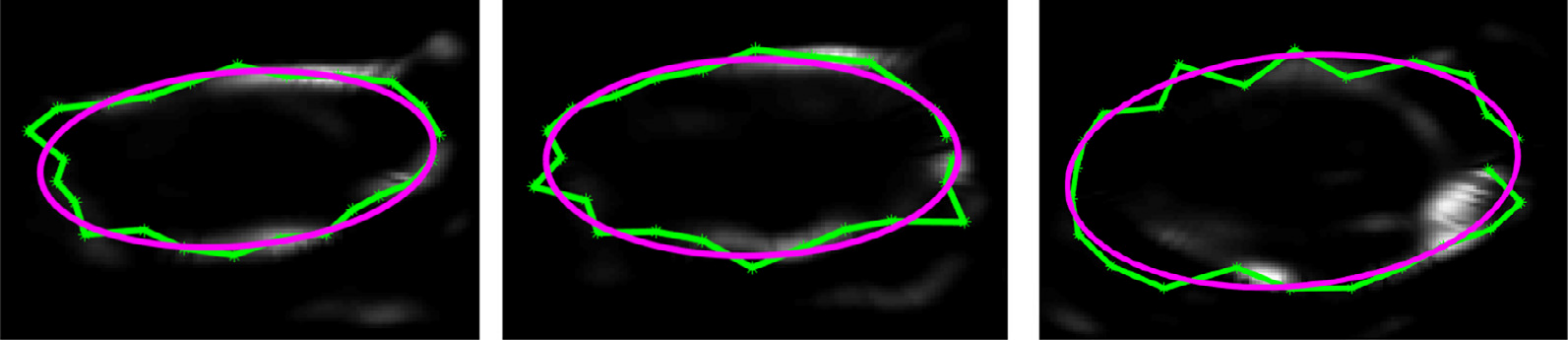
Example semi-axial images from one patient illustrating how fitting an ellipse (*magenta*) to the provisional contour obtained by finding peaks in the directional edge map (*green*) reduces the influence of outliers and smooths the contour.
4. **Projection of 2-D contours into 3-D:** The final step of the SE algorithm was to transform all of the 2-D uterine contours derived in image space back into their real space positions along the semi-axial planes defined during the manual initialization step. Each point contributing to an ellipse generated during the boundary regularisation step became a surface point in the 3-D uterine contour, as illustrated in Figure 7a. The final uterine segmentation was formed from a single conforming 3-D boundary around the surface points, which was generated via triangulation using the “boundary” function in MATLAB 2017a (The MathWorks, Natick, MA, USA), as illustrated in Figure 7b. Similarly to a conventional convex hull operation (Chazelle 1993), this function enveloped a set of surface points, but included an additional parameter called the “shrink factor,” which pulled the 3-D boundary towards the interior of the hull. This was important for ensuring a distinct boundary between the uterine head and the cervical body, particularly in cases where the uterine fundus was close to the cervix.

**Fig. 7:**
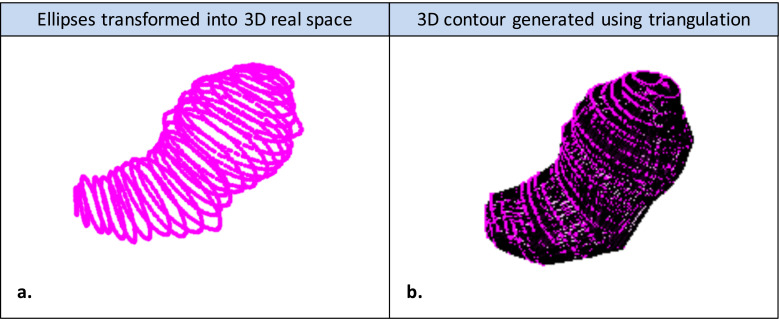
(a) Three-dimensional orientation of each individual elliptical contour generated on semi-axial ultrasound slices. (b) Visualization of final 3-D contour achieved using triangulation to envelop all 3-D surface points.

### Evaluation of algorithm performance

Three observers used the SE algorithm to semi-automatically segment the uterus on each of the 44 patient images included in the independent validation cohort. The Dice similarity coefficient (DSC) (Dice 1945) and mean absolute surface-to-surface distance (MSSD) (Yan et al. 2010) were measured between each algorithm-derived contour and the gold standard manual contour. For 3-D volumetric contours A and B, the DSC was calculated as (2|A∩B|)/(|A|+|B|), with 1 representing perfect overlap and 0 representing no overlap, and the MSSD was defined as the mean absolute distance between every point on the surface of A (n points total) and the nearest neighbouring point on the surface of B, as outlined in the equation

(4) $$\text{MSSD}=\frac{1}{n}\sum_{i=1}^{n}∥A_{i}-B_{i}∥$$

The median and interquartile range (IQR) of DSC and MSSD values from all three observers are reported (i) for each patient individually and (ii) over the study population as a whole.

To assess whether it would be possible to implement the SE algorithm on a clinically relevant time scale, the time required to complete the manual initialisation for each of the 44 US images was recorded for one observer (S.M.). The median and IQR time was reported. Additionally, the computation time for the automatic segmentation steps was also recorded.

## Results

### Training phase

**Fig. 8:**
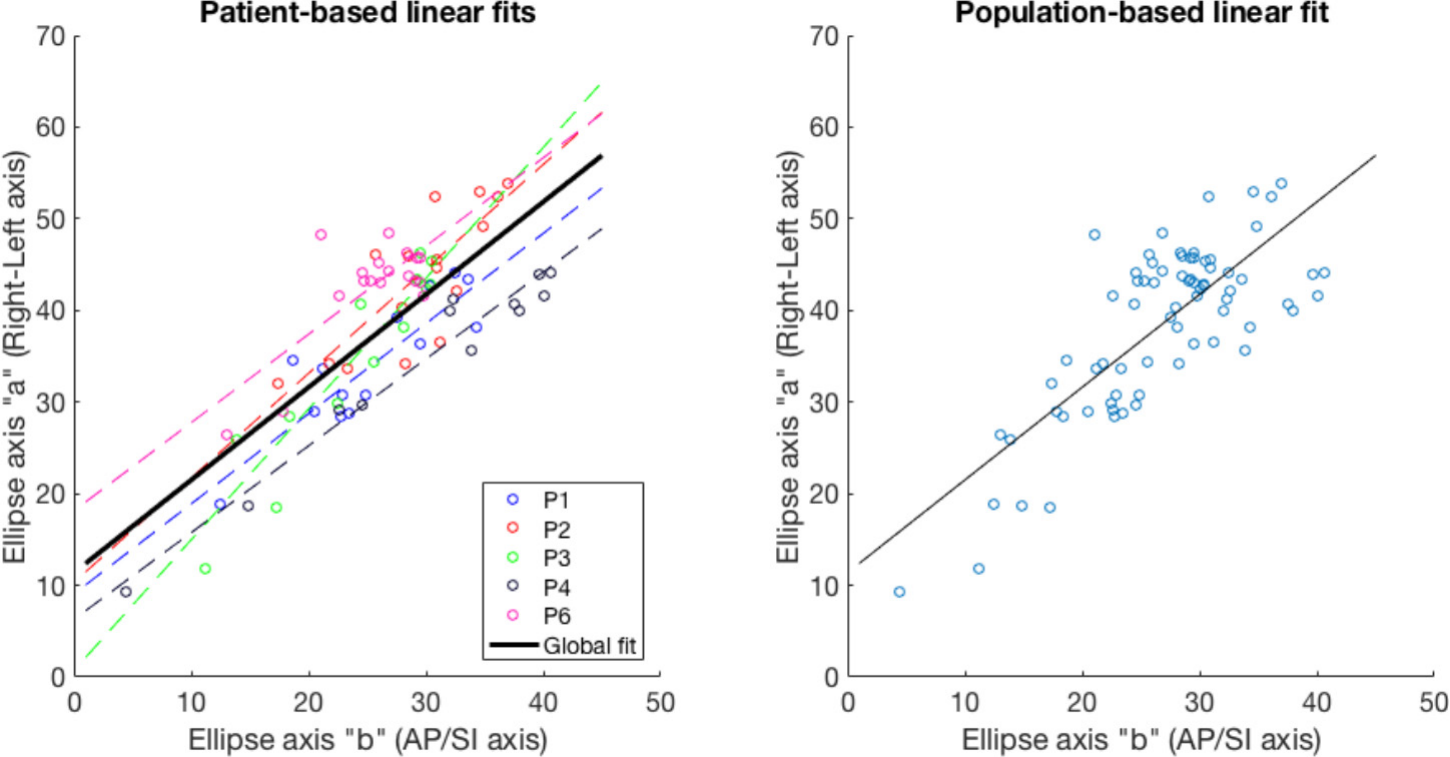
Relationship between ellipse axes for each patient individually (*dotted lines*) and globally (*black line*). Note: Patient-specific data were not used in the stacked-ellipse algorithm, but are provided to illustrate the inter-patient variability in uterine shape. The equation for the global linear fit was a = 1.01*b* + 11.3. On the right, the same data are shown, but without the patient-specific information for visual clarity. AP = anterior–posterior; SI = superior–inferior.

The relationship between the major and minor axes (axes a and b, respectively) from the ellipses providing the best fit to manually contoured semi-axial uterine cross sections is illustrated (i) for each of the five patients in the training cohort individually and (ii) for the entire population in Figure 8. The linear fit used to estimate axis a (the right–left extent of the uterus) from *b* was a = 1.01 * *b* +11.3. The coefficient of determination (*R*^2^) for this linear fit was 0.60.

### Segmentation phase

The SE algorithm was implemented for all patients in the test cohort using the parameters in Table 2. The parameters were selected based on previous experience in using the multiscale generalised gradient vector flow algorithm developed by Le et al. (2015) to segment the uterus of healthy volunteers on data acquired in a previous study (Mason et al. 2018).

**Table 2:** Values of user-tuneable parameters in the stacked-ellipse algorithm used for all segmentations

Parameter	Selected value
a (standard deviation of Gaussian smoothing kernel in eqn [2])	4
*k* (edge preservation parameter in eqn [3])	1
*r* (length of radial search region used for peak detection)	29

**Fig. 9:**
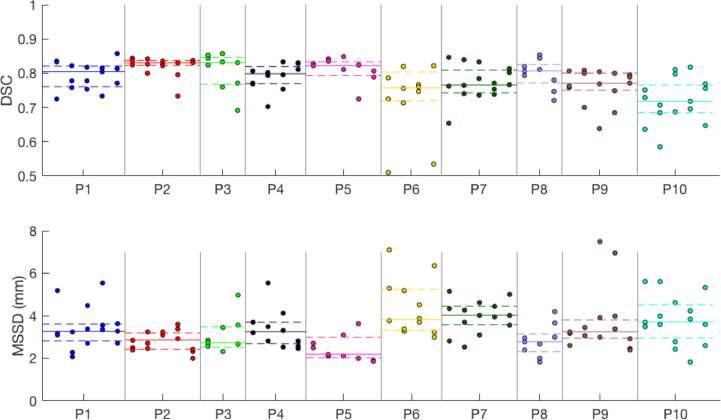
Dice similarity coefficient (DSC) and mean absolute surface-to-surface distance (MSSD) between each observer's use of the stacked-ellipse (SE) algorithm and the corresponding manual contour. Patients P1–P10 are represented in different colours and are separated by *vertical lines*. Columns represent ultrasound images from different time points. The three points in each column correspond to the result from each observer. The median and interquartile range for each patient are superimposed over the plots as *solid and dashed lines*, respectively.

The agreement between the SE algorithm from all three observers and the manual gold standard contours for the validation cohort is outlined in Table 3. Figure 9 illustrates these results graphically for each observer and each patient. The overall median [IQR] DSC and MSSD were 0.80 [0.03] and 3.3 [0.2] mm, respectively.

**Table 3:** Agreement between stacked-ellipse algorithm contours initialized by three observers and gold-standard 3-D manual contour of the uterus

Patient	Median [interquartile range]	
	Dice similarity coefficient	Mean absolute surface-to-surface distance
1	0.80 [0.06]	3.3 [0.8]
2	0.83 [0.01]	2.9 [0.8]
3	0.83 [0.08]	2.7 [1.0]
4	0.80 [0.05]	3.3 [1.0]
5	0.82 [0.04]	2.2 [1.0]
6	0.76 [0.08]	3.8 [1.9]
7	0.77 [0.07]	4.0 [0.9]
8	0.81 [0.05]	2.8 [0.8]
9	0.77 [0.05]	3.2 [0.9]
10	0.72[0.08]	3.7 [1.6]
Cohort average	0.80 [0.03]	3.3 [0.2]

The median [IQR] time required for observer S.M. to perform the manual initialization was 40 [16] s. The computation times for the remaining steps of the SE algorithm, when implemented in MATLAB 2017a on a computer with a 2.8-GHz Intel Core processor and 16 GB of RAM, are summarized in Table 4.

**Table 4:** Example computation times required for each step of the stacked-ellipse algorithm per 2-D slice and for a representative uterine volume*

	Example computation time (s)	
	Per slice	Per volume (38-slice example)
Interpolation of 3-D ultrasound image onto 2-D semi-axial plane	3.4	129.2
Generation of directional edge map	0.01	0.38
Ellipse initialization	<0.01	0.29
Contour deformation (peak finding and boundary regularisation)	0.1	3.8
2-D to 3-D transformation	-	9
Total	-	142.7

⁎ All steps were implemented in MATLAB 2017a (The MathWorks, Natick, MA, USA) on a computer with a 2.8-GHz Intel Core processor and 16 GB of RAM.

## Discussion

**Fig. 10:**
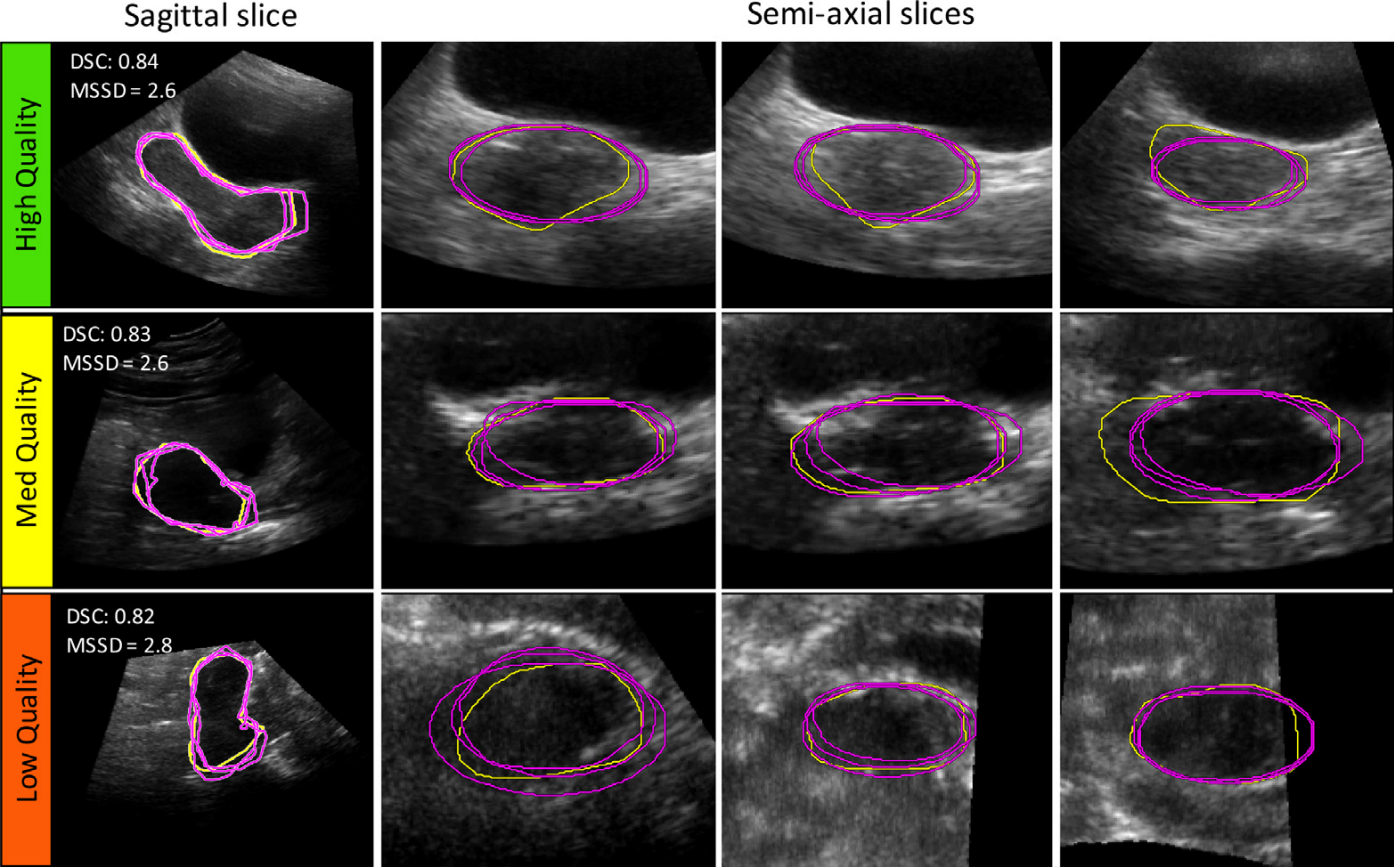
Example segmentations using the stacked-ellipse (SE) algorithm (*magenta,* three observers) compared with the corresponding gold standard manual segmentation (*yellow*). Each row contains example 2-D cross sections from the final 3-D segmentation in various orientations for high, medium and low ultrasound image quality. The Dice similarity coefficient (DSC) and mean absolute surface-to-surface distance (MSSD, mm) for each segmentation are displayed on the sagittal slice.

Previous work has indicated that the median [IQR] DSC and MSSD between manual contours drawn by different observers are 0.78 [0.11] and 3.20 [1.8] mm, respectively (Mason et al. 2017). These serve as benchmark values for assessing whether or not algorithm-derived segmentations can accurately determine the position and shape of the uterus. As the agreement between the SE algorithm segmentations and manual segmentations (median [IQR] DSC and MSSD of 0.80 [0.03] and 3.3 [0.2] mm, respectively) was within the range of inter-observer manual contour agreement, the SE algorithm was considered to have acceptable accuracy for segmenting the uterus prior to RT delivery. Unlike Elekta's Assisted Gyne Segmentation algorithm (Mason et al. 2017), there were no cases of complete failure (*i.e.,* complete geometric miss of the true uterine boundary or failure of the algorithm to generate a 3-D contour) when segmenting the uterus with the SE algorithm. Furthermore, the SE algorithm maintained a high segmentation accuracy when US image quality was poor and even when the US field of view did not completely cover the uterus, as illustrated in Figure 10.

The length of the major axis of elliptical uterine cross sections increased with increasing minor axis length. Although the least-squares linear fit describing this relationship slightly differed between patients in the training cohort, as illustrated in Figure 8a, the overall trend was similar enough to provide a good first approximation of the major axis given the minor axis. This was confirmed in the segmentation phase, where the linear relationship derived from a training cohort of only 5 patients was successfully applied to a completely different cohort of patients, in which the final segmentation result achieved the desired accuracy. To compare the overall trend between major and minor axes between the training and validation cohorts, the manual 3-D contours for the first US image available for patients in the validation cohort were parameterised as ellipses in the same way as they were in the training cohort, such that the elliptical axes lengths could be extracted. In the test cohort, the relationship between axes a and b was a = 1.3*b* +11.1, which is similar to the trend calculated for the training cohort (a = 1.01*b* + 11.3).

In current clinical practice, CBCT is commonly used to verify whether the uterus is inside or outside of the PTV. This process usually takes a few minutes, with poor-quality images requiring more time for analysis. The average time required for the manual initialisation step for the SE algorithm was under a minute, indicating that this algorithm could be implemented in a clinically acceptable time scale (using current practice in CBCT image analysis as a benchmark for what is considered “clinically acceptable”).

All subsequent steps used in the SE algorithm were not computationally expensive (and, therefore, not time consuming), except for the step where the 3-D US image was interpolated onto a series of arbitrarily orientated semi-axial planes. Without any optimisation, the computation time of this step ranged from 30 s to 3 min in MATLAB, depending on the number of semi-axial slices constituting the uterus. Although code optimisation and translation into a compiled language such as C could significantly reduce the algorithm run time, the time required to segment the uterus using the SE algorithm in its current form is on the order of a few minutes, which is considered clinically acceptable.

One limitation of this study is the small sample size; although these results indicate that the SE algorithm can accurately segment the uterus given a training cohort of 5 patients and a completely independent validation cohort of 10 patients from an entirely different hospital, a larger data set would be required to confirm the algorithm's performance. In particular, no patients were included in the analysis that had a Federation Internationale de Gynecologie Obstetrique (FIGO) cervical cancer stage greater than IIIB (range: IIA–IIIB, median: IIB, see Table 1 for baseline patient characteristics). As stage IV cervical cancers often manifest as bulky tumours that have heterogenous soft tissue contrast, the assumptions of uterine shape and contrast made by the SE algorithm may not be valid in this population. However, as the incidence of stage IV cervical cancers in the United Kingdom is relatively low (8% of cases as reported by Cancer Research UK [2017]), only a small proportion of the population is likely to be unsuitable for the SE algorithm in its current form.

Although the SE algorithm is accurate to the level of inter-observer contour agreement, one aspect of the algorithm that could potentially be improved is the trade-off between prior knowledge of uterine shape and feature extraction. The assumption that uterine cross sections are elliptical in shape was strictly imposed. Although this successfully constrained the segmentations in cases where the true uterine boundary is obscured or otherwise unclear, it came at the cost of preventing the contour from conforming to boundaries that deviated from this elliptical shape, as illustrated in Figure 10 by the discrepancies between the manual (*yellow*) and algorithm-derived (*magenta*) contours. Future work could investigate the use of 3-D boundary regularisation methods, more complicated shape priors (such as the super-ellipses described by Gong et al. [2004]) or an additional weighting parameter to modify the contour flexibility based on US image quality. Alternatively, it may be possible to segment the uterus on 2-D semi-axial slices generated during the manual initialization step of the SE algorithm using machine learning approaches such as support vector machines (Yang et al. 2011) or neural networks (Egmont-Petersen et al. 2002; Carneiro et al. 2012; Ronneberger et al. 2015), whereby each pixel in an image is classified as either “uterus” or “background.” This is appealing because assumptions about target shape and contrast do not necessarily have to be explicitly taken into account; rather, a database of images and corresponding gold standard segmentations would be used to establish the model parameters (*i.e.,* support vectors in a support vector machine or weights in a neural network) that best classify pixels into foreground or background. However, a major drawback of these approaches is the large amount of training data needed to generate a database representative of the entire target population, which prohibited the investigation of these methods in this study.

Finally, the images analysed in this study were generated by the Clarity Autoscan, which employs a simple (*i.e.,* non-compounding) 3-D sector-scan format that is not necessarily optimised for imaging the uterus for purposes of image-guided RT. Future work should test whether performance of uterine boundary segmentation methods such as the SE algorithm can be further improved by improvements in uterine image quality using techniques such as 3-D extended aperture compounding (Mason et al. 2018).

## Conclusions

The agreement between contours derived from the SE algorithm and manual contours was equal to inter-observer manual contour agreement of the uterus. Though it is unclear whether the SE algorithm could be adapted to segment the uterus in cervical cancer patients with bulky disease, these results indicate that it is accurate when used in patients with FIGO stage IIIB or lower disease. Furthermore, the SE algorithm segmented the uterus in a clinically relevant time scale, and used a small training set to provide the prior knowledge needed for uterine shape used during the initialization phase. Though confirmation of the algorithm performance is needed in a larger patient cohort, the results from this work indicate that the SE algorithm could be implemented in an adaptive RT workflow to quickly and accurately segment the uterus on 3-D US images.
